# 

**Published:** 1893-04

**Authors:** 


CONTENTS

Vol. 40. No. 4.

| | PAGE. |
|---|---|
| The Advancement of Women | 73 |
| Tobacco | 75 |
| Behind the Counter | 78 |
| A Chapter on Wrinkles | 80 |
| Infectious Diseases | 82 |
| Town and Country | 84 |
| Severed Fingers Reuniting | 86 |
| Hurried Dinners | 87 |
| Keeping Fruit and Vegetables | 88 |
| Docking Horses' Tails | 88 |
| How to Improvise a Vapor Bath | 89 |
| Care of the Baby | 89 |
| The Fatal Camel | 90 |
| The Future Life | 90 |
| Superstitious Remedies | 91 |
| Miscellaneous | 92 |
| Literary | 96 |
| Flats and Flats | 96 |

INDEX TO ADVERTISEMENTS OF HALF A PAGE AND OVER.

| | PAGE. |
|---|---|
| Dr. Jaeger's Sanitary Woolens | I |
| National Mutual Insurance Co. | II |
| The Press Claims Co. | III |
| Club Press | IV |
| Hall's Journal Premiums | VII |
| The World Almanac | VIII |
| J. Fehr's Compound Talcum | IX |
| Herba Vita | X |
| Royal Baking Powder | XI |
| Buffalo Lithia Water | XI |

